# Transverse colonic volvulus presenting in a 19-year-old female with subsequent sigmoid volvulus

**DOI:** 10.1093/jscr/rjae556

**Published:** 2024-08-28

**Authors:** Jennifer Stiene, Meghan Barber, Francisco Rodriguez Silva, Sean J Halloran, Joseph J Sferra

**Affiliations:** University of Toledo College of Medicine and Life Sciences, 3000 Arlington Ave, Toledo, OH 43614, United States; University of Toledo College of Medicine and Life Sciences, 3000 Arlington Ave, Toledo, OH 43614, United States; University of Toledo College of Medicine and Life Sciences, 3000 Arlington Ave, Toledo, OH 43614, United States; University of Toledo College of Medicine and Life Sciences, 3000 Arlington Ave, Toledo, OH 43614, United States; Department of Surgery, ProMedica Health System, 2147 N Cove Blvd, Toledo, OH 43606, United States

**Keywords:** volvulus, transverse colonic volvulus, transverse colon

## Abstract

Transverse colonic volvulus is exceptionally rare and is the rarest compared to sigmoid or cecal volvulus. This case report summarizes the care of a young 19-year-old woman who presented with transverse colonic volvulus. This woman came to the emergency room with abdominal pain, nausea, and vomiting, and she had no risk factors for a volvulus. This case report has the goal of raising awareness among those taking care of anyone coming in for abdominal pain. Volvulus is a serious issue and can be life threatening if not treated appropriately.

## Introduction

Transverse colonic volvulus is exceptionally rare and even more rare in teenagers and young adults. In a systematic review conducted by Huerta *et al.* [1], 317 cases (0.94%) of transverse colonic volvulus were found in patient data over 90 years. They also found that it was more common in patients in their third or fourth decade of life. We present a 19-year-old female who was diagnosed with transverse colonic volvulus with no history of abdominal surgery.

## Case report

**Figure 1:**
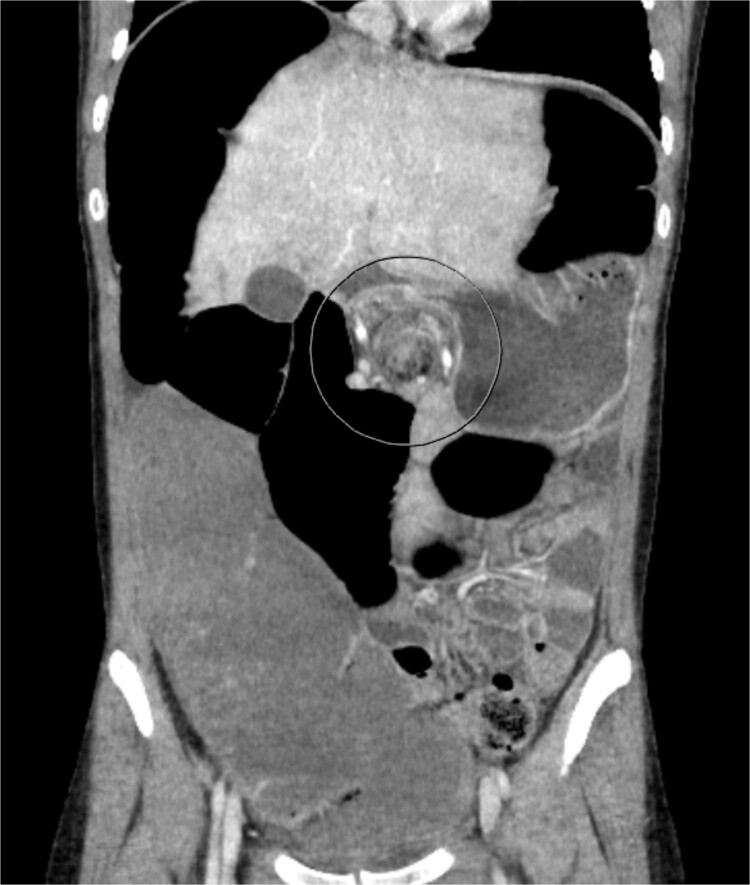
CT of the abdomen performed on Day 1 of admission. Shows colonic distension with volvulus at the splenic flexure.

**Figure 2:**
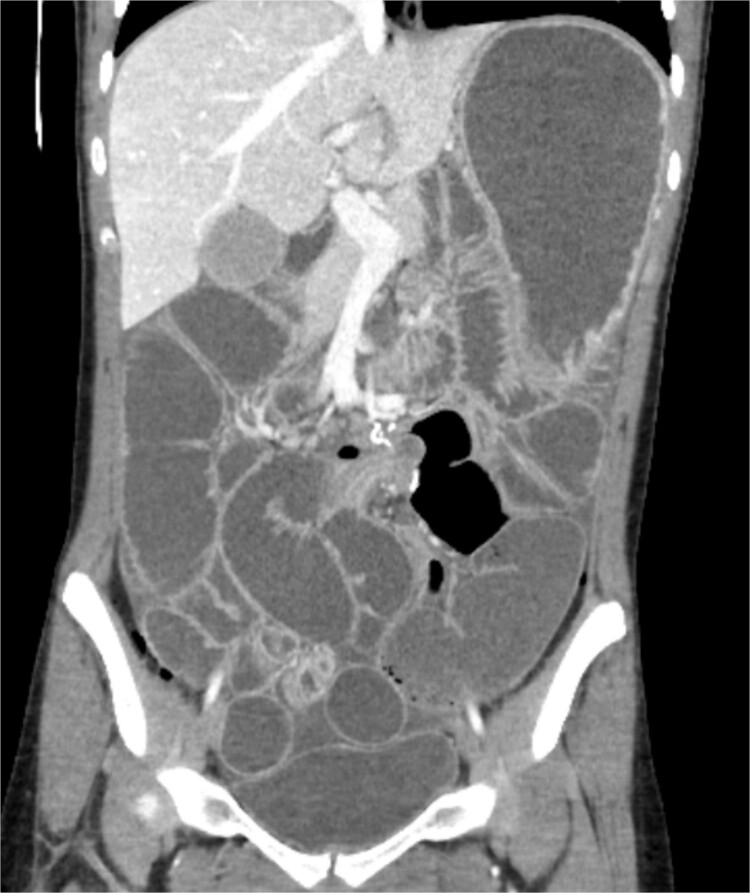
CT of the abdomen performed 8 days post operation. Shows proximal dilatation of the small bowel.

A 19-year-old female presented to the emergency department with complaints of epigastric abdominal pain, nausea, vomiting, and having passed no gas or bowel movements for 4 days. Her past medical history consisted of irritable bowel syndrome, gastroparesis, depression and anxiety, eosinophilic esophagitis, and opioid abuse. She did not take any medication for her depression and anxiety. Her past surgical history included upper and lower endoscopy and surgical ablation of genital warts. She has a significant family history of a brother who has ulcerative colitis. Upon physical exam, her abdomen was tympanic and distended. There was epigastric tenderness with no guarding or rebound tenderness. Due to concern for a bowel obstruction, a nasogastric tube was placed, and a computed tomography (CT) scan was done (Fig. 1). Imaging showed immense distension of the colon due volvulus at the splenic flexure with narrowing of the mesenteric vasculature and free fluid in the abdomen. The patient was taken for an exploratory laparotomy emergently with a preoperative diagnosis of a large bowel obstruction due to internal hernia versus volvulus. During the operation, it was found that the distal transverse colon was the point of obstruction and showed scarring with dilatation proximal to the narrowing, and there was no internal hernia identified. An extended right colectomy was performed with ileocolic anastomosis. She had a return of bowel function on postoperative Day 4. Six days post-operation, the patient began to have nausea and vomiting and had not had a bowel movement in 2 days. Eight days post-operation, a CT scan was performed that illustrated proximal small bowel dilatation (Fig. 2). With concerns of another bowel obstruction, an exploratory laparotomy was performed. During the operation, a dilated small bowel and a twisted sigmoid colon were found consistent with a large bowel obstruction secondary to sigmoid volvulus. A completion colectomy with end ileostomy was performed.

## Discussion

Transverse colonic volvulus is a rare explanation for a large bowel obstruction due to its anatomical protective factors, such as the fixation of the hepatic and splenic flexures, and shorter mesentery [2]. When it does occur, it most commonly involves the splenic flexure, which is a watershed region at risk for ischemia.

Risk factors for colonic volvulus in general are surgeries involving bowel translocation, malignancy, and malrotation [2]. Chilaiditi syndrome is also specifically correlated with predisposing patients to transverse colonic volvulus [3]. Chilaiditi syndrome is a benign congenital defect where a segment of intestine lies between the liver and the diaphragm. A case report was identified and found the Chilaiditi syndrome was diagnosed in a 47-year-old male with an acute intestinal bowel obstruction that was found to be of the transverse colon on exploratory laparotomy [3]. Another risk factor for transverse colonic volvulus is constipation. The extensive dilatation of the transverse colon puts it at risk for future twisting [2, 4]. Our patient did have a diagnosis of irritable bowel syndrome with constipation possibly contributing to her presentation.

It is critical to diagnose quickly to prevent the need for resection of the large bowel. The mortality rate in the case of transverse colonic volvulus is 33% [2, 5]. Due to its rarity and high mortality rate, it is at the utmost importance for surgeons to be aware of this and keep it on their differential even in a younger patient.
